# Canine Distemper Virus Infection Leads to an Inhibitory Phenotype of Monocyte-Derived Dendritic Cells *In Vitro* with Reduced Expression of Co-Stimulatory Molecules and Increased Interleukin-10 Transcription

**DOI:** 10.1371/journal.pone.0096121

**Published:** 2014-04-25

**Authors:** Visar Qeska, Yvonne Barthel, Vanessa Herder, Veronika M. Stein, Andrea Tipold, Carola Urhausen, Anne-Rose Günzel-Apel, Karl Rohn, Wolfgang Baumgärtner, Andreas Beineke

**Affiliations:** 1 Department of Pathology, University of Veterinary Medicine Hannover, Hannover, Germany; 2 Center for Systems Neuroscience, Hannover, Germany; 3 Department of Small Animal Medicine and Surgery, University of Veterinary Medicine Hannover, Hannover, Germany; 4 Unit for Reproductive Medicine, Small Animal Clinic, University of Veterinary Medicine Hannover, Germany; 5 Department of Biometry, Epidemiology and Information Processing, University of Veterinary Medicine Hannover, Hannover, Germany; Indian Institute of Science, India

## Abstract

Canine distemper virus (CDV) exhibits a profound lymphotropism that causes immunosuppression and increased susceptibility of affected dogs to opportunistic infections. Similar to human measles virus, CDV is supposed to inhibit terminal differentiation of dendritic cells (DCs), responsible for disturbed repopulation of lymphoid tissues and diminished antigen presenting function in dogs. In order to testify the hypothesis that CDV-infection leads to an impairment of professional antigen presenting cells, canine DCs have been generated from peripheral blood monocytes *in vitro* and infected with CDV. Virus infection was confirmed and quantified by transmission electron microscopy, CDV-specific immunofluorescence, and virus titration. Flow cytometric analyses revealed a significant down-regulation of the major histocompatibility complex class II and co-stimulatory molecules CD80 and CD86 in CDV-infected DCs, indicative of disturbed antigen presenting capacity. Molecular analyses revealed an increased expression of the immune inhibitory cytokine interleukin-10 in DCs following infection. Results of the present study demonstrate that CDV causes phenotypical changes and altered cytokine expression of DCs, which represent potential mechanisms to evade host immune responses and might contribute to immune dysfunction and virus persistence in canine distemper.

## Introduction

Canine distemper is a worldwide occurring infectious disease of dogs, caused by a morbillivirus, closely related to measles virus (MV) [1], [2]. Similar to human measles clinical findings in canine distemper virus (CDV)-infected dogs include fever, rash, respiratory signs, and lymphopenia. Affected animals are prone to opportunistic infections as a consequence of generalized lymphoid depletion and profound immunosuppression [3], [4]. Moreover, persistent infection of peripheral lymphoid organs and the central nervous system of carnivores leads to long lasting immune alterations and immune mediated neuropathology [5], [6].

Dendritic cells (DCs) represent the most potent antigen presenting cell population, which initiate primary T cell responses and play an important role also for B cell immunity [7]. Several pathogens, including human herpesvirus type-1 as well as human and feline immunodeficiency viruses, target DCs and have evolved strategies to modulate their cytokine expression and antigen presenting capacity, thereby promoting virus immune evasion and persistence [8]–[10]. Other mechanisms include alteration of endocytosis, vesicle trafficking, and immunological synapse formation or apoptosis induction of infected DCs [11]–[15]. A disturbed function of antigen presenting cells, including DCs, is supposed to contribute to immunosuppression in measles patients [16]–[19]. Moreover, following infection of the respiratory tract, MV-infected DCs might mediate virus transmission to secondary lymphoid organs [7], [20]. During the chronic disease stage of canine distemper, cells with a DC-like morphology seem to serve as the primary host cells for the virus, which might promote viral persistence in lymphoid organs [21]. Thus, an inhibited terminal differentiation of DCs is currently discussed to be responsible for diminished antigen presenting function and disturbed repopulation of lymphoid tissues in CDV-infected dogs, as suggested for MV-infection [14], [21], [22], [23]. In addition, CDV-infection of thymic DCs may result in compromised T cell maturation, promoting the release of immature, potentially autoreactive lymphocytes, demonstrating a potential participation of DCs in both CDV-induced immunosuppression and immunopathology [21]. However, whether CDV has the ability to infect canine DCs and direct viral effects upon these professional antigen presenting cells have not yet been confirmed.

The aim of the present study was to determine the permissiveness of canine DCs to CDV *in vitro*. Besides antigen presentation via the major histocompatibility complex (MHC), adequate T cell activation by DCs requires co-stimulation by molecules such as CD80 (aka B7-1) and CD86 (aka B7-2). An additional signal is mediated by DC-released cytokines leading to T cell polarization (e.g. Th1 and Th2 responses) [10], [24]. Thus, in order to testify the hypothesis that infection leads to an impaired T cell stimulatory capacity of these cells, the impact of CDV upon molecules involved in antigen presentation and co-stimulation and the associated cytokine expression was investigated.

## Materials and Methods

### Generation of Monocyte-derived Dendritic Cells

Blood collection of dogs was approved and authorized by the local authorities (Niedersächsisches Landesamt für Verbraucherschutz und Lebensmittelsicherheit (LAVES), Oldenburg, Germany, permission number 13A303).

Monocyte-derived dendritic cells (moDCs) were generated as previously described, with minor modifications [25]–[27]. Briefly, 20 ml of fresh heparinized blood was taken from clinically healthy dogs (n = 16). Peripheral blood mononuclear cells (PBMC) were obtained by density gradient centrifugation (500×g) using Histopaque-1077 (Sigma-Aldrich, Germany) at room temperature for 30 minutes. PBMC were carefully collected from the interface, washed twice with phosphate buffered saline (PBS) +0.02% ethylenediaminetetraacetic acid (EDTA), counted and adjusted to a concentration of 2×10^6^ cells/ml, seeded in RPMI 1640 medium (PAA, Austria) supplemented with 100 UI/ml penicillin, 100 µg/ml streptomycin, and 10% fetal calf serum (FCS) and incubated for 24 hours at 37°C and 5% CO_2_. Subsequently, non-adherent cells were removed by gentle washing with PBS and adherent cells were incubated under standard conditions for additional six days, supplemented with 10.6 µg/ml recombinant human (rh) GM-CSF (R&D Systems, MN, USA) and 20 µg/ml recombinant canine (rc) IL-4 (R&D Systems, MN, USA). Fresh medium was added every third day. During medium change, non-adherent cells were collected, centrifuged, and supernatant (SNT) was discarded. Diluted cell pellet was transferred back to the culture flask.

To characterize DC generation *in vitro*, monocytes were analyzed at day one after cell adherence and non-adherent moDCs at day seven in culture by phase contrast microscopy, transmission electron microscopy, and flow cytometry (see below).

### Canine Distemper Virus Infection of Monocyte-derived Dendritic Cells

Non-adherent moDCs were harvested after the incubation period of seven days for the infection experiment. Cells were centrifuged and washed in RPMI 1640 medium (FCS free), seeded at the density of 0.25×10^4^ cells/ml in 6-well plates (Nunc, Sigma-Aldrich, Germany) and infected with the CDV strain R252 (kindly provided by Prof. S. Krakowka, Ohio State University, USA) at multiplicity of infection (MOI) 0.1. After an incubation period of two hours under standard conditions (37°C, 5% CO_2_), cells were centrifuged and washed twice with PBS to remove unbound virus. Subsequently fresh conditioned medium containing cytokines (rhGM-CSF, rcIL-4) and 10% FCS were added to the culture. Medium was changed every third day as described above.

Infection was determined by immunofluorescence (24, 72 and 120 hours post infection [hpi]), virus titration (120 hpi), reverse transcriptase polymerase chain reaction (120 hpi), and transmission electron microscopy (120 hpi). Phenotypical changes and cytokine expression of infected moDCs were analyzed by flow cytometry (120 hpi) and RT-qPCR (120 hpi), respectively.

### Phenotyping of Monocyte-derived Dendritic Cells by Flow Cytometry

Antibodies used for phenotypic analyses were either cell surface markers specific for dogs or cross-reacting with canine antigens [28]. For the characterization of antigen presenting cells (monocytes and moDCs, respectively), mouse monoclonal antibodies directed against CD1a (clone NA1/34-HLK, dilution of 1∶50; Abcam, United Kingdom), CD11c (clone CA11.6A1, dilution of 1∶6; AbD Serotec, United Kingdom), CD14 (clone TÜK4, conjugated with *R*-phycoerythrine, dilution of 1∶6; Abcam, United Kingdom), CD80 (clone CA24.5D4, dilution of 1∶6; CA24.5D4), CD86 (clone CA24.3E4, dilution of 1∶6), and MHC class II (clone CA2.1C12, dilution of 1∶6) were used. Antibodies against CD80, CD86 and MHC class II were kindly provided by Prof. P. Moore (University of California, USA). Immunoglobulin (Ig)G_1_ (AbD Setotec, United Kingdom) and IgG_2a_ (Southern Biotech, USA) were used as isotype controls. Adherent monocytes (one day in culture) and moDCs (seven days in culture) as well as CDV-infected and non-infected moDCs (120 hpi) were labelled as described [28]. Briefly, after the cultivation period cells were collected, centrifuged (250×g, 4°C, 10 minutes) and washed. Non-specific antibody binding was blocked by pretreatment of cells with 10 mg/ml human normal IgG (Globuman Berna, Switzerland). Primary antibodies and isotype controls were incubated for 30 minutes at 4°C and afterwards centrifuged (250×g, 20°C, 10 minutes) and washed twice with cell wash solution (BD Dickinson, Germany). Cells labeled with conjugated markers were resuspended in FACS flow solution (BD Dickinson, Germany) and stored at 4°C until use. Non-conjugated primary antibodies were incubated with the secondary antibody (goat anti-mouse pycoerythrin; Dianova, Germany) for 30 minutes at 4°C, followed by centrifugation (250×g, 20°C, 10 minutes) and two washing steps. Subsequently, cells were resuspended in FACS flow solution and analyzed immediately. Cells were gated using forward scatter height (FSC-H) and side scatter height (SSC-H) not exceeding 2% positive staining with serotypes.

Cell phenotyping was performed using the FACSCalibur flow cytometer (Becton Dickinson, USA) and data were analyzed with FlowJo software (Tree star, OR, USA).

### Immunofluorescence

For the detection of infected cells and quantification of the infectivity rate, respectively, cells were labeled using a monoclonal mouse anti-CDV-specific antibody (clone D110; kindly provided by Prof. A. Zurbriggen, University of Bern, Switzerland). Briefly, cells were transferred to a glass slide by cytospin centrifugation (250×g, 5 minutes). After fixation with paraformaldehyde (4%) for 30 minutes at room temperature, cells were washed with phosphate buffered saline Triton X (PBST). Non-specific blocking was performed with goat and horse serum (5% each) for 20 minutes. Subsequently, cells were incubated with the primary antibody (dilution 1∶100) for 4 hours at room temperature, followed by incubation with the secondary antibody (goat anti-mouse Cy3; 1∶100; Jackson, ImmunoResearch, Dianova, Germany) for 1 hour at room temperature in a dark chamber. For counterstaining cells were incubated with bisbenzimidine (1∶100; Sigma-Aldrich, Germany) for 15 minutes at room temperature. The percentages of CDV-infected cells were determined at 24, 72 and 120 hpi in duplicates by immunofluorescence microscopy (Olympus IX-70, Olympus Life Science Europe GmbH, Germany).

### Virus Titration

At 120 hpi, the cell free SNT of CDV-infected moDCs was harvested to calculate the 50% log^10^ tissue culture infectious dose/ml (TCID_50_/ml). Briefly, SNT was centrifuged at 300×g at 4°C, aliquoted and stored at −80°C until use. SNT was diluted logarithmically from 10^0^ to 10^−8^ in RPMI 1640 medium containing 10% FCS and titrated in 96-well microtiter plates (Nunc, Sigma-Aldrich, Germany) containing Vero.dogSLAM cells (1.5×10^4^ cells/well). After an incubation period of five days (120 hpi) under standard conditions cells were examined and evaluated for presence of cytopathogenic effects. The TCID_50_ was calculated as described [29], [30]. All samples were evaluated in triplicates.

### Transmission Electron Microscopy

Isolated monocytes at day one and generated moDCs at day seven in culture as well as CDV-infected moDCs at 120 hpi were centrifuged (100×g, 4°C, 10 minutes) and collected in 1.5 ml tubes. Subsequently cells were fixated with 2.5% glutaraldehyde and incubated overnight at 4°C. Post-fixation was performed in 1% aqueous osmium tetroxide and after five washes in cacodylate buffer (five minutes each) samples were dehydrated through series of graded alcohols and embedded in Epon 812 medium. Semi-thin sections were cut on a microtome (Ultracut Reichert-Jung, Leica Microsystems, Germany) and stained with uranyl citrate for 15 minutes. After eight washing steps samples were incubated with lead citrate for seven minutes. Ultra-thin sections were cut with a diamond knife (Diatome, USA) and transferred to copper grids. Samples were examined by a transmission electron microscope (EM 10C, Zeiss, Germany).

### Lactate Dehydrogenase Assay

For detecting cell lysis, a lactate dehydrogenase assay (LDH; CytoTox 96 Non-radioactive Cytotoxicity Assay, Promega, USA) was performed according to manufacturer’s instructions. Briefly, after centrifugation of cells, SNT from non-infected and CDV-infected cells at 120 hpi were carefully collected and stored at −80°C until use. 50 µl of SNT and 50 µl of substrate mix were added to a 96-well microtiter plate and incubated for 30 minutes. Reaction was stopped with 1 M acetic acid (stop solution) and absorbance was measured at 490 nm using an ELISA reader (Fluorostat Optima, BMG Labtech, Germany). Data were analyzed using the Optima data analysis software (BMG Labtech, Germany).

### Cytokine Expression Analyses and Virus Quantification by Reverse Transcriptase-Quantitative Polymerase Chain Reaction

Primers for the generation of standards and primers for measuring the quantity of specific cytokines and CDV are listed in Table S1. PCR primer sequences for detecting glyceraldehyde-3-phosphate dehydrogenase (GAPDH), elongation factor-1α (EF-1α), hypoxanthine-guanine phosphoribosyltransferase (HPRT), tumor necrosis factor-α (TNF-α), transforming growth factor-β (TGF-β), interleukin (IL)-2, IL-6, IL-8, and IL-10 as well as for CDV were taken from the literature [31]–[36]. All primers were purchased from Eurofins MWG Operon (Ebersberg, Germany).

Total RNA was isolated from cultured cells at 120 hpi using the RNeasy Mini Kit (Qiagen, Germany) according to the manufacturer’s instructions. For the generation of serial standards dilutions, total RNA was extracted from the canine macrophage cell line DH82 (for GAPDH, EF-1α, TGF-β, TNF-α, IL-6, IL-8, and IL-10), persistently CDV-infected DH82 cells (for CDV and HPRT), and a canine lymph node (for IL-2) using TRIZOL (Invitrogen, Germany). RNA concentrations were calculated by measuring the optical density at 260 nm (GeneQuant pro, Amersham Biosciences Europe GmbH, Germany). Subsequently, total RNA was reversely transcribed to complementary DNA using the Omniscript Kit (Qiagen, Germany) with RNase Out (Invitrogen, Germany) and Random Primers (Promega, Germany) following the manufacturer’s instructions.

For the production of standards, PCR was performed using a Biometra TProfessional basic thermocycler (Biometra GmbH, Germany), as described before [33]–[35]. Annealing temperature was adjusted to 50°C (IL-2), 56°C (HPRT), 57°C (TGF-β), 58°C (TNF-α, IL-6), 59°C (GAPDH, IL-10, CDV), and 60°C (EF-1α, IL-8) for two minutes and amplification was achieved using AmpliTaq DNA Polymerase (Applied Biosystems, USA) in 1× GeneAmp PCR Buffer II (Applied Biosystems), with 1.25 mmol/L MgCl_2_, 0.2 mmol/L dNTP mix (Biosystems, USA), and 300 nmol/L of each primer. Polymerase chain reaction products of standards were subsequently analyzed by agarose gel electrophoresis and extracted using NucleoSpin Extract II Kit (Macherey-Nagel, Germany) for production of a standard dilution from 10^2^ to 10^8^ copies per microliter.

Reverse transcriptase-quantitative polymerase chain reaction (RT-qPCR) and data analysis were performed using the Mx3005P QPCR System (Agilent Technologies, Germany) [33]–[35], [37]. In addition to the standard dilution, complementary DNA of samples and negative controls were measured in duplicate on the same run. Quantification was carried out in 25 µl of Brilliant SYBR Green qPCR Core Reagent Kit (Agilent Technologies, Germany). Amplification was performed using 0.05 U/µl SureStart Taq DNA Polymerase in 1× Core PCR buffer with 2.5 mmol/L (CDV, GAPDH, TNF-α, IL-6, IL-10) or 5 mmol/L (EF-1α, HPRT, IL-2, IL-8, TGF-β) MgCl_2_, 8.0% glycerol, 3% dimethyl sulfoxide (4% for TGF-β, IL-2), 150 nmol/L of each primer (Table S1), 30 nmol/L Rox as reference dye, and 200 µmol/L dNTP mix. Specificity of the products was assessed by melting curve analysis. Calculated copy numbers of each gene were normalized to an amount of 100 ng of transcribed RNA and gene expression values were normalized against the three housekeeping genes, GAPDH, EF-1α, and HPRT, using the software geNorm (Ghent University Hospital Center for Medical Genetics; available at http://medgen.ugent.be/~jvdesomp/genorm/) [38]. In brief, the software detects the most stable reference genes of which the geometric means were used to calculate a normalization factor for the genes of interest.

### Statistical Analyses

To determine the distribution of data a Shapiro-Wilk test and visualization assessment were performed. For not normally distributed values a non-parametric test (Mann-Whitney U-test) was used. For normally distributed data a student’s T-test was performed. For statistical analyses and visualization of data the SPSS software (IBM, USA) was used.

## Results

### In vitro Generation and Characterization of Monocyte-derived Dendritic Cells

#### Morphology

**Figure 1:**
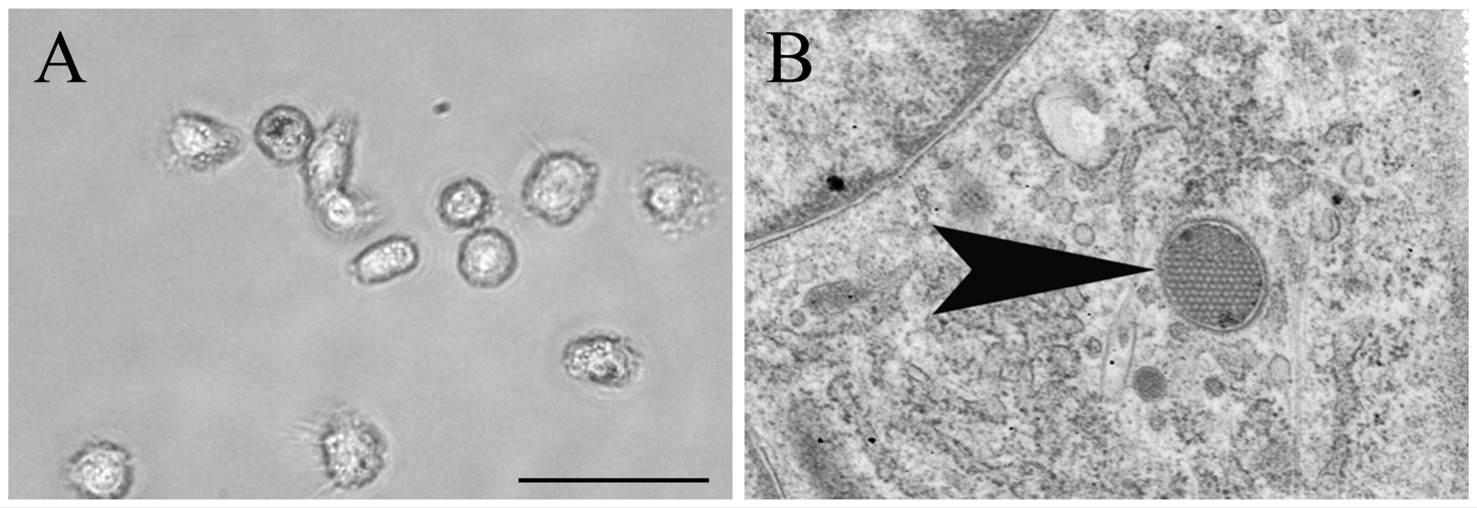
Morphological characterization of canine monocyte-derived dendritic cells at seven days in culture. A) Cultured cells showing a typical dendritic cell-like morphology with long cytoplasmic processes. Phase contrast microscopy, bar size = 20 µm. B) Periodical microstructure (wasp nest-like structure; arrowhead) in the cytoplasm representing a distinct feature of canine monocyte-derived dendritic cells. Transmission electron microscopy, magnification = 25.000×.

After seven days in culture in the presence of rcIL-4 and rhGM-CSF, the majority of isolated PBMC showed a typical DC-like morphology with long cytoplasmic processes, as demonstrated by phase contrast microscopy (Figure 1). moDC differentiation *in vitro* was confirmed by transmission electron microscopy which revealed a typical DC-like morphology, including long cytoplasmic processes, abundant Golgi apparatus formation, and only few lysosomes. In addition, periodical microstructures representing a distinct ultrastructural feature of canine moDCs [26] were found in cells at seven days in culture (Figure 1).

#### Phenotypical properties

**Figure 2:**
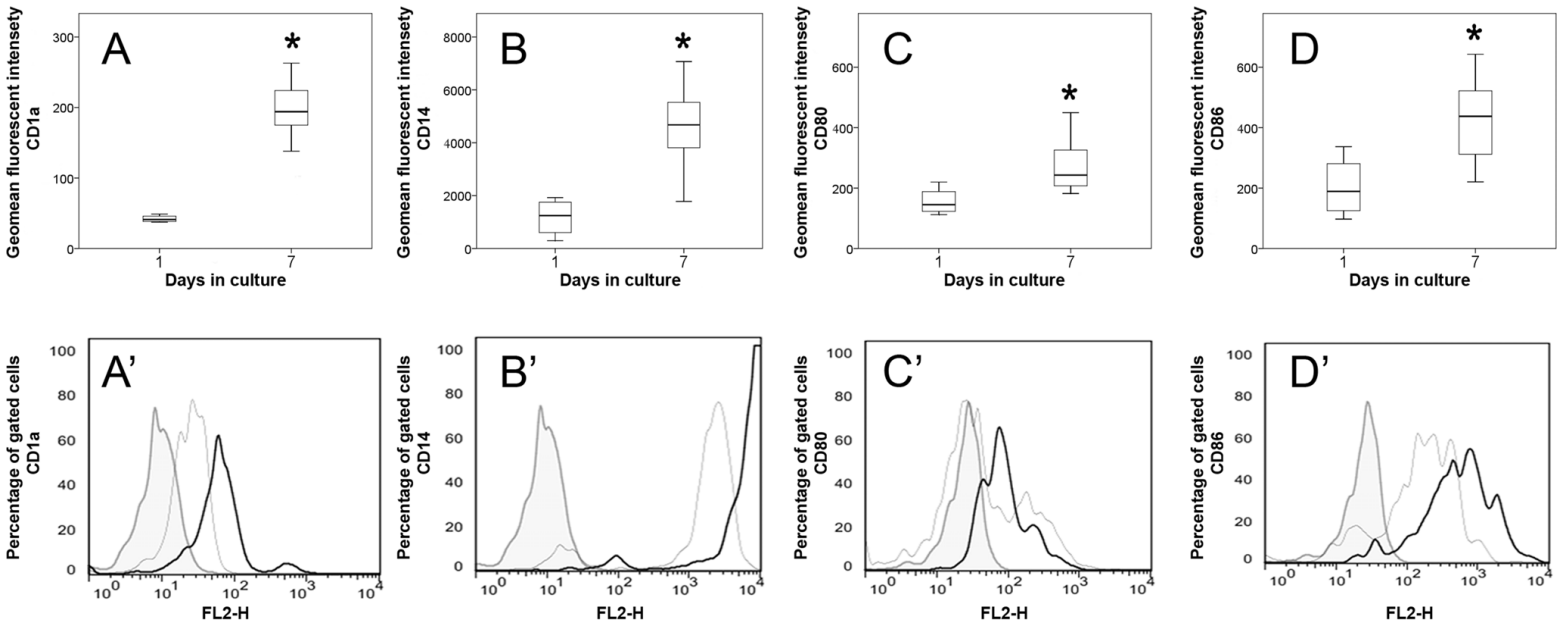
Phenotypic analyses of monocyte-derived dendritic cells by flow cytometry. Significantly increased (*; p≤0.05) expression of A) CD1a and B) CD14 and up-regulation of co-stimulatory molecules C) CD80 and D) CD86 at day seven compared to cells at day one in culture. Box and whisker plots display median and quartiles with maximum and minimum values. Representative histograms of A’) CD1a, B’) CD14, C’) CD80 and D’) CD86 expression intensity in gated cells. Filled tinted curve = isotype control; thin line = monocytes at day one in culture; thick black line = dendritic cells at seven days in culture.

Phenotypical analyses of PBMC (day one) and moDCs (day seven) were performed by flow cytometry. The percentage of gated cells was determined to characterize the phenotype of cells and the geometrical mean fluorescent intensity (GMFI) for the quantification of surface marker expression of monocytes and moDCs, respectively. The majority of cultured cells at day one and day seven expressed CD14 and CD11c, indicative of monocytic origin [39]. Noteworthy, in contrast to human beings and mice, canine moDCs do not lose the ability to express CD14 during cultivation [40]. An increased percentage of cells expressing the co-stimulatory molecule CD86 at day seven compared to day one in culture was noticed (p = 0.031), while no statistical differences were found for CD1a, CD11c, CD14, CD80 and MHC class II (data not shown). Analysis of GMFI revealed a significant up-regulation of the co-stimulatory molecules CD80 (p = 0.012) and CD86 (p = 0.018) as well as an increased surface expression of CD1a (p = 0.001) and CD14 (p = 0.001) of cells at day seven in culture compared to cells at day one in culture (Figure 2), indicative of DC differentiation [27], [40]. These generated moDCs were used for subsequent infection experiments *in vitro* (see below).

### Canine Distemper Virus Infection of Monocyte-derived Dendritic Cells

#### Virus detection by immunofluorescence, reverse transcriptase-quantitative polymerase chain reaction and virus titration

**Figure 3:**
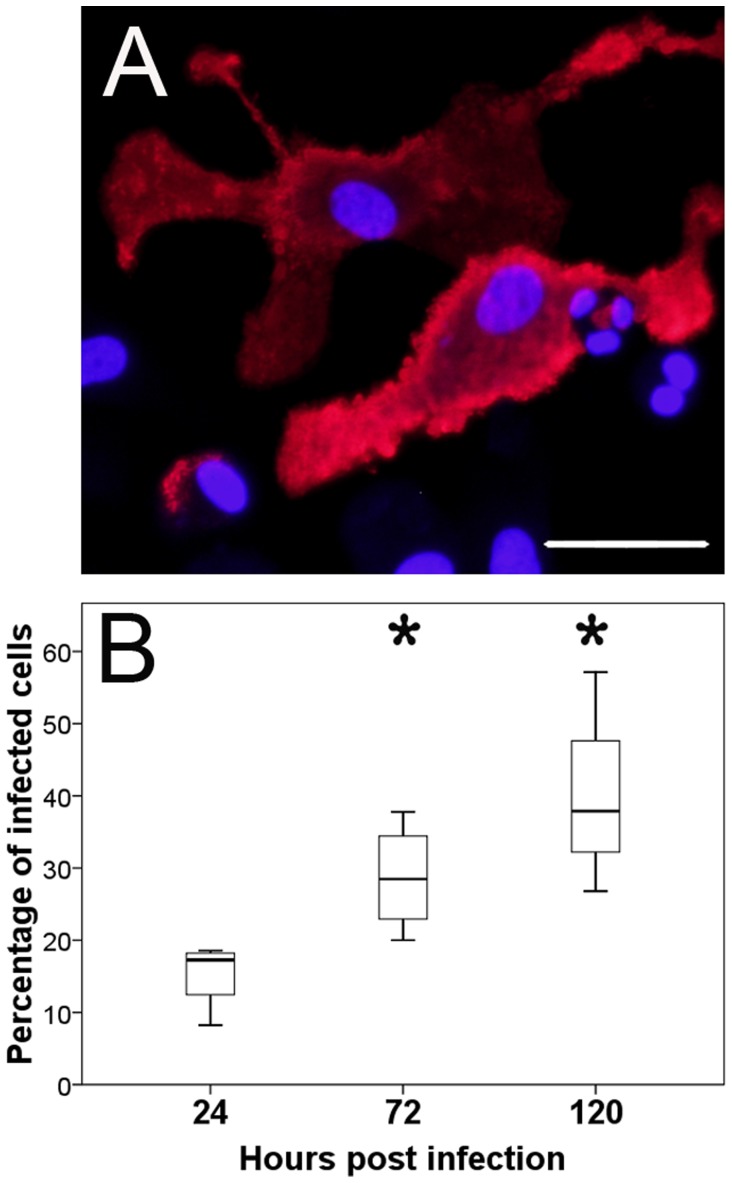
Detection of canine distemper virus (CDV) in monocyte-derived dendritic cells by immunofluorescence. A) Infected monocyte-derived dendritic cells at 72 hours post infection (hpi) labeled with a CDV-specific antibody (red color). Nuclear staining with bisbenzimidine (blue color), bar size = 20 µm. B) Quantification of CDV-infected cells revealed a significant increase (*; p≤0.05) at 72 and 120 hpi. Box and whisker plots display median and quartiles with maximum and minimum values.

In order to determine the ability of CDV to infect canine DCs and to quantify the infectivity rate, moDCs were infected at seven days in culture. Infections were stopped at 24, 72, and 120 hpi and cell cultures were investigated by immunofluorescence (Figure 3). CDV-infected cells were detected at 24 hpi. Following, significantly increased numbers of infected cells were found at 72 hpi (p = 0.031) and 120 hpi (p = 0.023; Table 1) compared to 24 hpi, indicative of a time-dependent increase of the infectivity rate (Figure 3).

**Table 1:** Comparison between canine distemper virus-infected and non-infected cells.

Parameter	Result*
CDV protein	**p = 0.023↑**
CDV RNA	**p = 0.001↑**
TCID_50_	**p = 0.001↑**
CD1a	p = 0.295
CD11c	p = 0.364
CD14	p = 0.456
CD80	**p = 0.018↓**
CD86	**p = 0.036↓**
MHC class II	**p = 0.029↓**
IL-2	p = 0.351
IL-4	p = 0.887
IL-8	p = 0.058↑*
IL-10	**p = 0.041↑**
TNF-α	p = 0.111
TGF-β	p = 0.193

*results of statistical analyses (p-values) at 120 hours post infection; bold values display significant changes compared to control (non-infected cells), ↑ = significantly increased; ↓ = significantly decreased compared to control; ↑* = statistical tendency of increase compared to control; CDV = canine distemper virus; RNA = ribonucleic acid; MHC = major histocompatibility complex; IL = interleukin; TNF = tumor necrosis factor; TGF = transforming growth factor; TCID_50_ = tissue culture infectious dose 50.

CDV-RNA within infected moDCs was detected at 120 hpi by RT-qPCR (Table 1). In addition, the amount of cell free virus in the SNT at 120 hpi (median value = 247.0 TCID_50_/ml, minimum value = 31.6 TCID_50_/ml, maximum value = 1778.3 TCID_50_/ml) was determined by virus titration demonstrating the presence of infectious virus particles and productive infection, respectively (Table 1).

#### Transmission electron microscopy and lactate dehydrogenase assay

**Figure 4:**
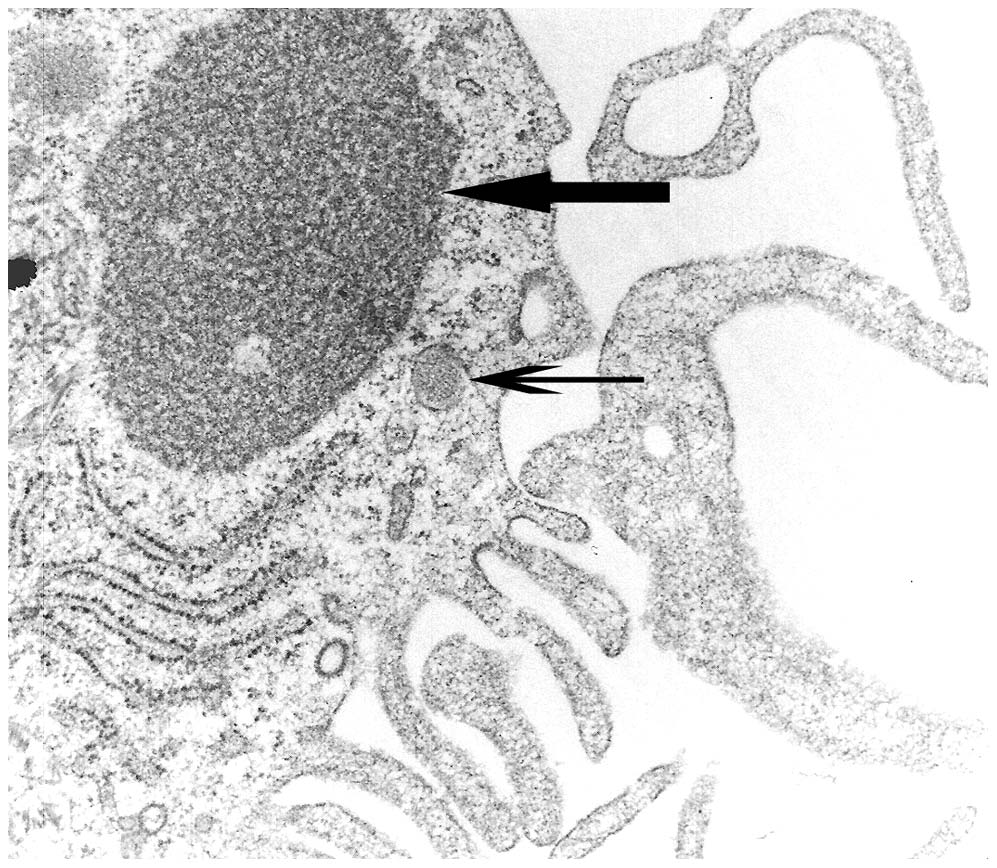
Ultrastructural analysis of canine distemper virus-infected monocyte-derived dendritic cells. Accumulation of viral nucleocapsid in the cytoplasm (arrow). Note also periodical microstructure (arrowhead) and cytoplasmic processes representing features of dendritic cells. Transmission electron microscopy, magnification = 50.000×.

CDV-infection was confirmed by electron microscopy, revealing the presence of virus nucleocapsid in the cytoplasm of moDCs (Figure 4). However, despite the presence of virus particles, ultrastructural examination showed neither cytoplasmic or nuclear degenerative changes nor necrosis or apoptosis of infected cell cultures at 120 hpi.

The virtual lack of virus-induced cytopathogenic effects was further confirmed by the lactate dehydrogenase assay, which revealed no differences between infected and non-infected cells at 120 hpi (Table 1).

#### Flow cytometry

**Figure 5:**
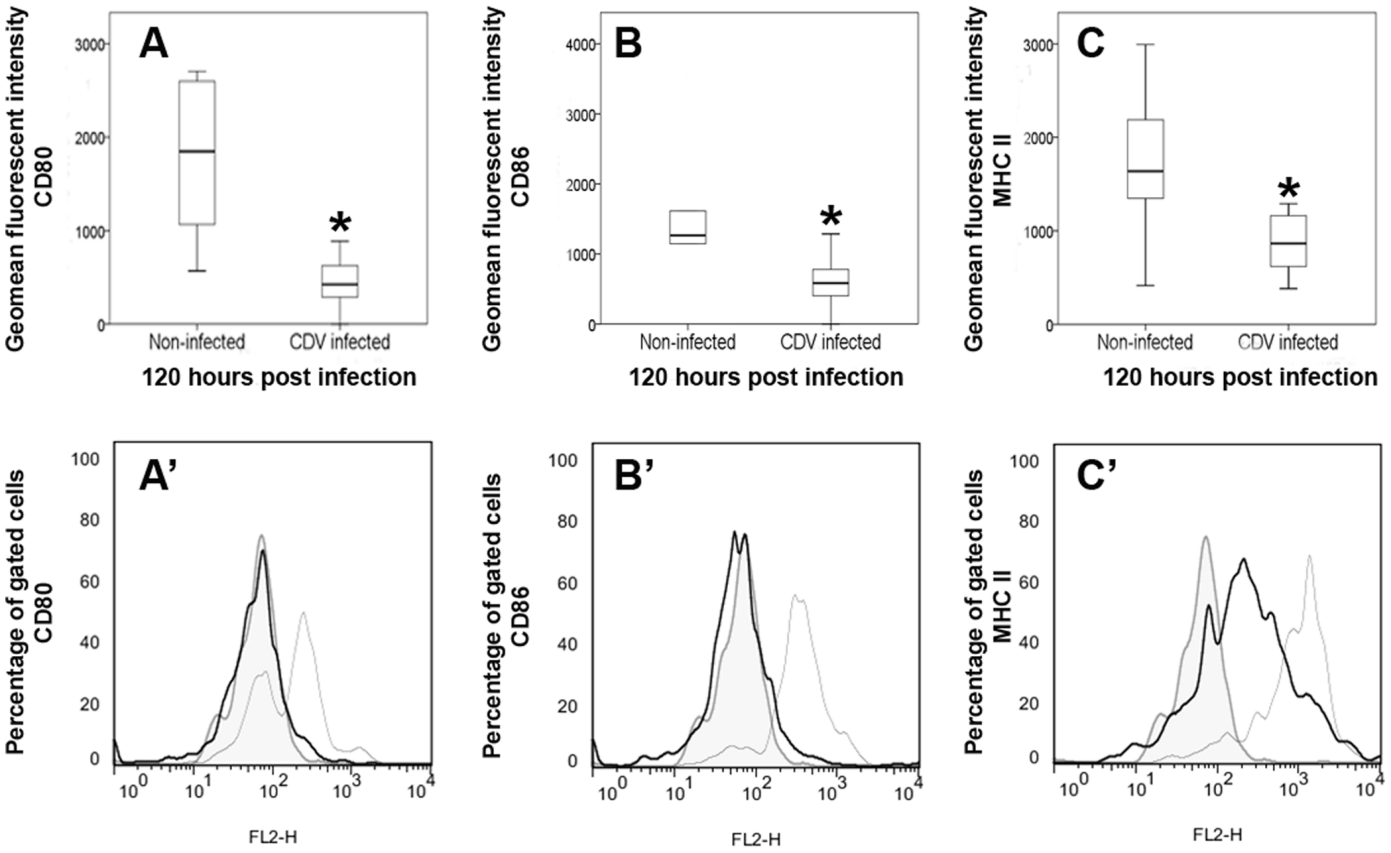
Phenotypic analyses of canine distemper virus-infected monocyte-derived dendritic cells by flow cytometry. Significantly decreased (*; p≤0.05) expression of A) CD80, B) CD86 and C) major histocompatibility complex (MHC) class II of infected cells compared to non-infected cells at 120 hours post infection. Box and whisker plots display median and quartiles with maximum and minimum values. Representative histograms of A’) CD80, B’) CD86, C’) MHC class II expression intensity in gated cells. Filled tinted curve = isotype control; thin line = non-infected cells; thick black line = infected cells.

In order to determine the impact of CDV upon phenotypical properties of canine DCs, infected and non-infected cells were evaluated at 120 hpi by flow cytometry. Indicative of a reduced antigen presenting and co-stimulatory function, analyses revealed a significant down-regulation of MHC class II (p = 0.029) and of the co-stimulatory molecules CD80 (p = 0.018) and CD86 (p = 0.036) of CDV-infected moDCs compared to non-infected moDCs (Figure 5). The expression of other surface markers (CD1a, CD11c, CD14) showed no differences between infected cells and non-infected controls (Table 1).

#### Cytokine expression analyses

**Figure 6:**
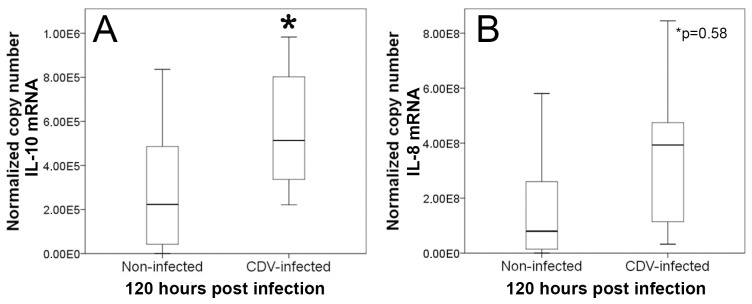
Cytokine expression analyses of canine distemper virus-infected monocyte-derived dendritic cells. Reverse transcriptase-quantitative polymerase chain reaction revealed a significantly increased (*; p≤0.05) interleukin-10 (IL-10) mRNA-expression and a statistical tendency (*p = 0.058) of an increased interleukin-8 (IL-8) transcription in infected cells compared to non-infected cells at 120 hours post infection. Box and whisker plots display median and quartiles with maximum and minimum values.

To substantiate the hypothesis that CDV-infection together with reduced MHC class II, CD80 and CD86 expression lead to an impaired immunogenic capacity of moDCs, respectively, cytokine expression was quantified by RT-qPCR. Molecular analyses revealed a significantly increased transcription of the anti-inflammatory and inhibitory cytokine IL-10 in CDV-infected moDCs (Figure 6). Moreover, a statistical tendency of an increased transcription of IL-8 (Figure 6), which is involved in chemotaxis and leukocyte recruitment in viral diseases [41], was observed following *in vitro* infection. Other cytokine mRNA levels (IL-2, IL-6, TNF-α, TGF-β) showed no differences between infected and non-infected cells at 120 hpi (Table 1).

## Discussion

The present study demonstrates the ability of CDV to infect canine DCs and to modulate their antigen presenting properties and cytokine expression which has the potential to influence host innate and adaptive immune responses. Many viruses, including MV, have developed strategies to alter antigen presentation or co-stimulatory properties of DCs in order to evade host immune responses, thereby causing immunosuppression and an increased susceptibility to opportunistic infections [12], [42]–[46]. Based on the present data, a similar mechanism is suggested for CDV-infection of dogs. The observed CDV-induced DC modulation might represent a mechanism to suppress protective immunity, which favors persistent infection in infected dogs. In agreement with this idea, it was reported that CDV-infected DC-like cells within lymphoid organs occur in advanced stages of canine distemper [21]. Furthermore, the virtual lack of detectable cytopathogenic or lytic effects as determined by phase contrast microscopy, electron microscopy, and LDH assay might contribute to virus spread within the organism via circulating DCs (Trojan horse strategy) as described for MV [47]. Similarly, restricted viral infection of CNS cells together with prevention of cytolysis causes limited recognition by the immune surveillance, which favors viral persistence and transmission within the brain in canine distemper [21], [48]–[53]. *In vitro* experiments have demonstrated that virulent CDV strains (e.g. strain A75/17) exhibit conformational properties of the F protein, which limit cell-to-cell fusion activity and prevent cytopathogenicity, Thus, persistent infection with delayed production of infectious virus might be a consequence of reduced spread of less fusogenic viruses compared to cytolytic CDV strains (e.g. strain Onderstepoort) [54]–[56]. In addition, human cytomegalovirus, murine cytomegalovirus, and Epstein-Barr virus infect and manipulate DCs to circumvent cell death, which causes persistent infection [12]. Besides this, disturbed function and prolonged survival of DC in canine distemper might compromise T cell maturation and selection, promoting the release of immature, potentially autoreactive cells as discussed for canine distemper [21], [57], [58].

The present study shows, that CDV-infection down-regulates MHC class II and co-stimulatory molecules (CD80 and CD86) of DCs which could have the ability to impair T cell activation in affected dogs. Previous studies revealed an inhibition of antigen presenting cells in canine distemper as a consequence of reduced IL-1 production and increased prostaglandin E_2_ release [59]. Thus, results of the present study further support the hypothesis that disturbed antigen presenting function contributes to reduced mitogen-induced lymphocyte proliferation observed in CDV-infected dogs [56]–[62]. Moreover, diminished T helper cell function as a consequence of impaired antigen presentation in persistently infected dogs might lead to disturbed germinal center and plasma cell formation and reduced class switch from IgM to IgG in canine distemper [63]. Dysregulation of antigen presenting properties is supposed to account for immunosuppression in human measles, e.g. by down-regulation of IL-12 by DCs, which leads to a failure to activate T cells [44], [64], [65]. However, the precise role of DC infection in the pathogenesis of natural MV-infection remains to be determined since differing effects have been described, probably attributed to varying DC maturation states *in vitro* and species-specific properties (e.g. human versus rodent), respectively [24]. Similar to findings of the present study, MV-infection of mice causes a down-regulation of co-stimulatory molecules as well as of MHC class I and II molecules in DCs [16]–[18]. While mature immunogenic DCs express high levels of MHC class II, CD80, and CD86, reduced expression of these surface molecules together with an increased expression of IL-10 is a hallmark of semi-mature DCs, which exhibit tolerogenic or inhibitory properties [66]. Moreover, induction of IL-10 by infected DCs is supposed to suppress Th1 and prolong Th2 immune responses in measles patients, which leads to ineffective immunity [64], [67]. IL-10 production suppresses host immune responses and facilitates the ability of intracellular pathogens to escape the host innate immune defense [68]. For instance, this cytokine has been demonstrated to reduce antiviral immunity in a variety of persistent infectious diseases, such as acquired immunodeficiency syndrome (AIDS), hepatitis C, Theiler’s murine encephalomyelitis, and lymphocytic choriomeningitis [69]–[73]. Thus, IL-10 is currently discussed as a target for therapeutic approaches in chronic viral diseases, which might also apply for canine distemper. IL-10 favors also systemic infection and neuroinvasion as shown in mice experimentally infected with West Nile virus [74]. The inhibitory effect of the cytokine is mediated by exhaustion and anergy of virus-specific T cells and induction of suppressive Foxp3^+^ regulatory T cells [75]–[77]. Furthermore, IL-10 has been shown to inhibit DC maturation and function, e.g. by reducing the density of co-stimulatory molecules, demonstrating that the described DC impairment *in vitro* might have been induced partly also in a paracrine manner.

In conclusion, for the first time CDV-infection of canine moDCs has been demonstrated *in vitro.* Modulation of antigen presenting MHC and co-stimulatory molecules of CDV-infected DCs might contribute to immune dysfunction in affected dogs. However, further studies are needed to understand the functional relevance of CDV-mediated DC alterations in the pathogenesis of immunopathology and virus persistence in canine distemper. Getting insights into the interaction between viruses and DCs is fundamental to understand the pathogeneses of infectious disorders, which has implication for prevention (e.g. vaccination) and novel treatment strategies [78].

## Supporting Information

Table S1 Gene expression analyzed by polymerase chain reaction. S = sense; AS = antisense; bp = base pair; EF-1α = elongation factor-1α; GAPDH = glyceraldehyde-3-phosphate dehydrogenase; HPRT = hypoxanthine-guanine phosphoribosyltransferase; IL = interleukin; TGF-β = transforming growth factor-β; TNF-α = tumor necrosis factor-α.
(DOCX)
