# Supplementary material for: Canine Distemper Virus Infection Leads to an Inhibitory Phenotype of Monocyte-Derived Dendritic Cells In Vitro with Reduced Expression of Co-Stimulatory Molecules and Increased Interleukin-10 Transcription
Source: PLoS One. 2014 Apr 25;9(4):e96121. doi: 10.1371/journal.pone.0096121 (PMC4000198; doi:10.1371/journal.pone.0096121)
Supplement: Table S1 — Gene expression analyzed by polymerase chain reaction. S = sense; AS = antisense; bp = base pair; EF-1α = elongation factor-1α; GAPDH = glyceraldehyde-3-phosphate dehydrogenase; HPRT = hypoxanthine-guanine phosphoribosyltransferase; IL = interleukin; TGF-β = transforming growth factor-β; TNF-α = tumor necrosis factor-α. (DOCX) [file pone.0096121.s001.docx]

Supplemental Table S1. Gene expression analyzed by polymerase chain reaction

| **Qualitative polymerase chain reaction** | | | **Real time quantitative polymerase chain reaction** | | |
| --- | --- | --- | --- | --- | --- |
| **Gene /**  **primer direction** | **Primer sequence** | **Genebank accession no., position** | **Gene /**  **primer direction** | **Primer sequence** | **Genebank accession no., position** |
| GAPDH S | AAGGTCGGAGTCAACGGATT | AB038240, 7-26 | GAPDH S | GTCATCAACGGGAAGTCCATCTC | AB038240, 196-218 |
| GAPDH AS | GCAGAAGAAGCAGAGATGATG | AB038240, 371-351 | GAPDH AS | AACATACTCAGCACCAGCATCAC | AB038240, 279-257 |
| EF-1α S | AGCCCTTGCGCCTGCCTCTC | X03558, 784-803 | EF-1α S | CAAAAACGACCCACCAATGG | AY195837, 770-789 |
| EF-1α AS | CAGACACATTCTTGACATTGAAGC | X03558, 1002-979 | EF-1α AS | GGCCTGGATGGTTCAGGATA | AY195837, 837-818 |
| HPRT S | TAAAAGTAATTGGTGGAGAT | CFU16661, 2-21 | HPRT S | GAGATGACCTCTCAACTTTAACTGAAAA | CFU16661, 17-44 |
| HPRT AS | ATTATACTGCGCGACCAAG | CFU16661, 123-105 | HPRT AS | GGGAAGCAAGGTTTGCATTG | CFU16661, 105-86 |
| IL-2 S | ACCTCAACTCCTGCCACAAT | D30710, 14-33 | IL-2 S | CCAACTCTCCAGGATGCTCAC | D30710, 196-216 |
| IL-2 AS | GCACTTCCTCCAGGTTTTTG | D30710, 302-283 | IL-2 AS | TCTGCTAGACATTGAAGGTGTGTGA | D30710, 276-252 |
| IL-6 S | TCTCCACAAGCGCCTTCTCC | U12234, 68-87 | IL-6 S | TGATGCCACTTCAAATAGTCTACCA | U12234, 156-180 |
| IL-6 AS | TTCTTGTCAAGCAGGTCTCC | U12234, 385-366 | IL-6 AS | TCAGTGCAGAGATTTTGCCGAGGA | U12234, 244-221 |
| IL-8 S | ACTTCCAAGCTGGCTGTTGC | U10308, 10-29 | IL-8 S | AAGAACTGAGAGTGATTGAC | D28772, 184-203 |
| IL-8 AS | GGCCACTGTCAATCACTCTC | U10308, 181-162 | IL-8 AS | TTTATACACTGGCATCGAA | D28772, 149-130 |
| IL-10 S | CCTGGGTTGCCAAGCCCTGTC | U33843, 235-255 | IL-10 S | GGTGGGAGCCAGCCGACACCAG | U33843, 49-70 |
| IL-10 AS | ATGCGCTCTTCACCTGCTCC | U33843, 446-427 | IL-10 AS | AAGAAGATCTTCACCCACCCGAAGG | U33843, 168-144 |
| TNF-α S | CCAAGTGACAAGCCAGTAGC | Z70046, 32-51 | TNF-α S | GGAGCTGACAGACAACCAGCTGA | Z70046, 133-155 |
| TNF-α AS | TCTTGATGGCAGAGAGTAGG | Z70046, 305-286 | TNF-α AS | GGAAGGGCACCCTTGGCCCT | Z70046, 223-204 |
| TGF-β S | AAGAAAAGTCCGCACAGCAT | NM_001003309, 430-450 | TGF-β S | TGGCGCTACCTCAGCAACCG | NM_001003309.1, 592-611 |
| TGF-β AS | CAGGCAGAAGTTAGCGTGGT | NM_001003309, 1026-1006 | TGF-β AS | AGCCCTCGACTTCCCCTCCA | NM_001003309.1, 706-687 |
| CDV S | ACAGGATTGCTGAGGACCTAT | AF378705, 769-789 | CDV S | GCTCTTGGGTTGCATGAGTT | AF378705, 954-973 |
| CDV AS | CAAGATAACCATGTACGGTGC | AF378705, 1055-1035 | CDV AS | GCTGTTTCACCCATCTGTTG | AF378705, 1036-1017 |

S = sense; AS = antisense; bp = base pair; EF-1α = elongation factor-1α; GAPDH = glyceraldehyde-3-phosphate dehydrogenase; HPRT = hypoxanthine-guanine phosphoribosyltransferase; IL = interleukin; TGF-β = transforming growth factor-β; TNF-α = tumor necrosis factor-α.
